# Cushing’s syndrome with no clinical stigmata – a variant of glucocorticoid resistance syndrome

**DOI:** 10.1186/s40842-018-0072-5

**Published:** 2018-12-18

**Authors:** Ved V. Gossain, Mohammad El-Rifai, Preethi Krishnan, Bhavini Bhavsar

**Affiliations:** 1 Division of Endocrinology, Department of Medicine, College of Human Medicine, Michigan State University, 788 Service Road, B323 Clinical Center, East Lansing, MI 48824 USA; 2 Department of Endocrinology, Licking Memorial Hospital, 1272 W Main Street, Newark, OH 43055 USA; 3 PeaceHealth Southwest Medical Center Diabetes Endocrine & Nutrition, 400 NE Mother Joseph Place, Vancouver, WA 98664 USA

**Keywords:** Cushing’s syndrome, Cortisol resistance, Cortisol resistance syndrome

## Abstract

**Background:**

Cortisol resistance syndrome is a very rare condition characterized by high cortisol levels, but without any clinical features of Cushing’s syndrome. Our objective is to present such a case.

**Case presentation:**

A 41 year old female presented with mild hirsutism and elevated urinary cortisol levels. Plasma cortisol levels were elevated and were not suppressed by conventional doses of dexamethasone on multiple occasions, but decreased following administration of higher doses of dexamethasone. Adrenocorticotropic hormone (ACTH) levels were inappropriately elevated. Despite significantly elevated cortisol levels, she did not develop any clinical signs or symptoms of Cushing’s syndrome. Pituitary and adrenal imaging did not reveal any abnormalities. Genetic testing for human glucocorticoid receptor did not reveal any mutations.

**Conclusions:**

Although we were not able to identify any new mutations, we believe that our patient has a variant of cortisol resistance syndrome. This syndrome should be considered in the differential diagnosis of patients who present with high levels of cortisol but have no clinical features of Cushing’s syndrome.

## Introduction

Cushing’s syndrome, due to hypercortisolism, is a well-recognized clinical entity with easily identifiable clinical features. [1] However, sometimes patients present with hypercortisolism without any clinical features of Cushing’s syndrome. This situation presents a diagnostic challenge for the clinicians. In such cases, besides making sure that there is no lab error in the assay of cortisol, the diagnostic possibilities include pseudocushing’s, cyclic Cushing’s syndrome, and cortisol resistance syndrome. The latter is a rare syndrome characterized by high cortisol levels with no clinical stigmata of Cushing’s syndrome and has been attributed to mutations in the human glucocorticoid receptor (hGR) gene. [2] Only about 30 separate probands have been reported so far. [3] We report a case of glucocorticoid resistance, who did not have a recognizable mutation of hGR. Although we were not able to identify any new mutations, we believe that our patient has a variant of the glucocorticoid resistance syndrome.

## Case report

A 41-year-old female was referred to the Michigan State University (MSU) endocrinology clinic in 2008 for elevated 24-h urinary free cortisol which was presumably obtained to evaluate hirsutism and irregular menstrual cycles. She had increased facial hair for more than 20 years, which she had managed cosmetically with intermittent waxing. She attained menarche around age 13, and had regular menstrual cycles till her early 20s, at which time she started to have irregular periods and was told she had polycystic ovarian syndrome. She has two children. The first child was conceived spontaneously and the second pregnancy required clomiphene therapy. Other medical history included hypothyroidism following radioactive iodine treatment for a toxic thyroid nodule and depression.

On physical examination, her blood pressure was (122/80 mmHg) and body mass index (BMI) was 22.6 Kg/m^2^, both normal. She had mildly increased facial hair on her chin and a palpable thyroid nodule in the right lobe. The remainder of the physical exam was normal, and she did not have any clinical features of Cushing’s syndrome.

On laboratory evaluation, 24-h urinary cortisol levels were 317 mcg (normal 3.5–45 mcg /day). Even though she didn’t have any signs or symptoms of Cushing’s syndrome elevated urinary cortisol levels required further evaluation. Repeated overnight and 48 h dexamethasone suppression tests (DST) failed to suppress cortisol levels and 24-h urine cortisol levels were persistently elevated at multiple times. (Table 1).

**Table 1:** Laboratory parameters in a patient with crs

Date of the test	24-Hr Urinary Cortisol [3.5–45 mcg/day]	Serum Cortisol AM: [4.3–19.8 mcg/dl] PM: [3.1–15.0 mcg/dl]	ACTH [10-60 pg/ml]	1 mg DST Cortisol Level [mcg/dl] (< 1.8 mcg/dl)	48 Hour DST Cortisol level [mcg/dl] < 1.8 mcg/dl	Testosterone [< 20–80 ng/dl]	DHEA-S [35–430 mcg/dl]
10/2008^a^	317						
12/2008				20.6			
02/2009					Baseline: 26.1 After: 12.1		
03/2009	320			14.3			
04/2009						136	278
09/2010		AM: 26.2				164	266
10/2010	133						
11/2010		AM: 23					
12/2010		AM: 30.6 Midnight: 21.8	53				330
01/2011							281
04/2011	89						
07/2012^b^		AM: 26.3 PM: 22.5				27	330
11/2012	120	AM: 24.8 PM: 24.8		19.9			
11/2013		AM: 25.9	53				
04/2014		AM: 21.1	60	20.5			
07/2014	164	AM: 19.2	38	6.4	10.4		
04/2015		AM: 23 PM: 21	68			39	303
05/2015	170						

*DST* Dexamethasone Suppression Test

*CRS* Cortisol Resistance Syndrome

^a^Age of Patient 41 years

^b^Age of Pateint 45 years

**Fig. 1:**
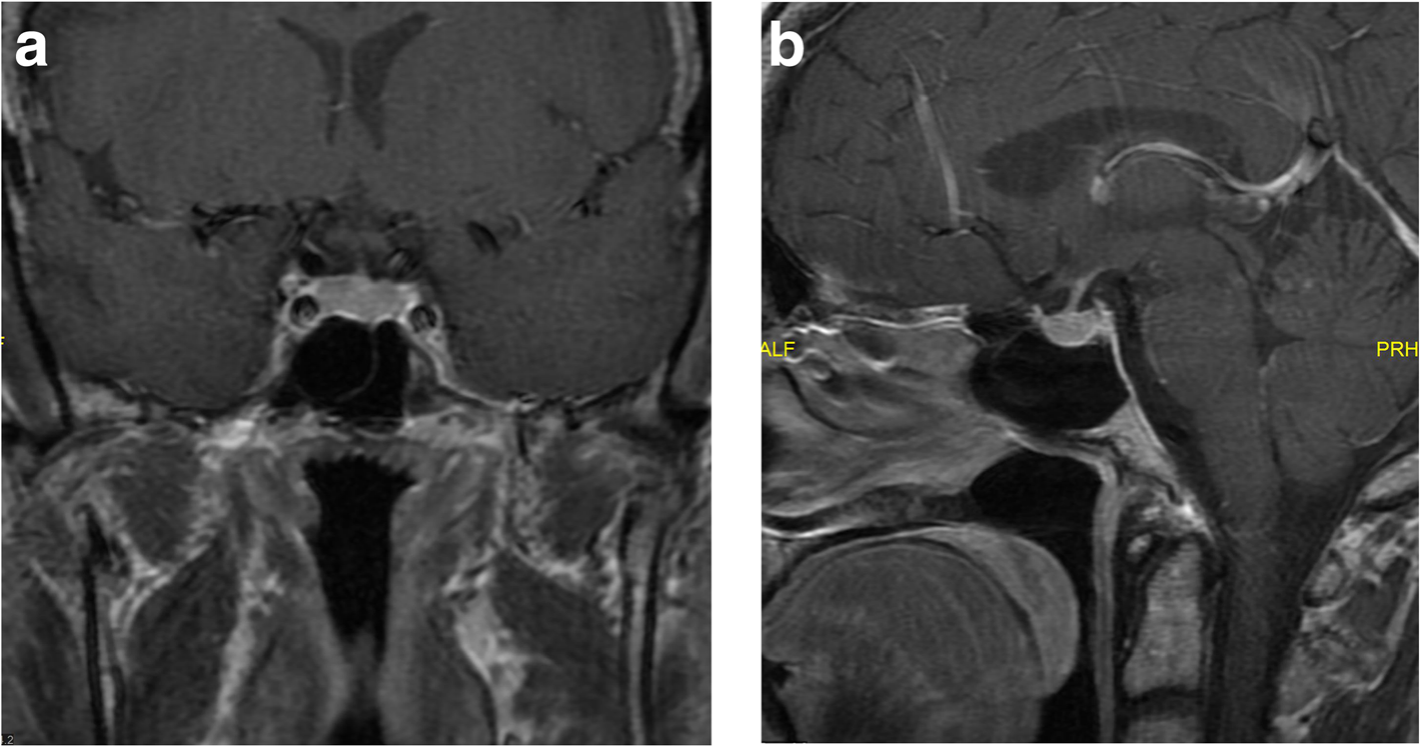
Pituitary MRI Coronal View. **a** Coronal view. **b** Sagittal view

ACTH levels were inappropriately normal, “too high” for elevated cortisol levels (Table 1) suggesting ACTH dependent hypercortisolism. Magnetic resonance imaging (MRI) of the pituitary was obtained, which did not show any abnormalities. (See Fig. 1 a and b).

Total testosterone levels were elevated with a level of 136 ng/dl (< 20–80) dehydroepiandrosterone sulfate (DHEA-S) levels were 278 mcg/dL (normal range 35.0–430.0). She was started on metformin with some improvement in hirsutism. Spironolactone was not given due to prior history of allergy. Pelvic ultrasound showed a simple cyst measuring 1.1 cm in the left ovary likely representing a follicle.

During the subsequent years she continued to have elevated cortisol levels with some nonspecific weight gain. Her blood pressure remained normal. Her hemoglobin A1C levels were normal and no clinical signs of Cushing’s syndrome developed over the next 10 years. She met with another endocrinologist for a second opinion who referred her for an Inferior petrosal sinus sampling. (Table 2) The sampling suggested pituitary source of ACTH. However, she was told that no further workup was needed till she developed clinical signs of Cushing’s. MRI of the abdomen was obtained that did not show adrenal mass or enlargement. A Dual-energy X-ray absorptiometry (DXA) obtained in May 2013, revealed osteopenia. A repeat bone mineral density (BMD) in December 2016 did not show any significant deterioration.

**Table 2:** Results of inferior petrosal sinus sampling in a patient with crs

Acth Levels [Pg/Ml]							
Base line levels				Levels after crh injections			
Sampling Sites	0 min	5 min	10 min	1 min	3 min	5 min	10 min
Right Inferior Petrosal Sinus	45	38	40	618	518	279	328
Left Inferior Petrosal Sinus	285	408	333	902	782	865	429
Peripheral Site	18	18	18	19	23	27	32

To determine if she had glucocorticoid resistance syndrome, genetic testing was performed (courtesy of Dr. Ameilia Sertedaki, Ph.D., Division of Endocrinology, First Department of Pediatrics, University of Athens Medical School, Athens, Greece 1152).

The coding regions of the human glucocorticoid receptor gene (hGR, *NR3C1*) were sequenced and showed no mutation. Her mother and daughters were tested for plasma cortisol levels, which were normal.

In order to determine if higher doses of dexamethasone will result in cortisol suppression, increasing doses of dexamethasone were administered (2, 4 and 8 mg) at night and plasma cortisol was measured the following morning. Plasma levels of dexamethasone were also obtained to confirm the patient had taken the prescribed doses of dexamethasone. The results are shown in Table 3.

**Table 3:** Cortisol level with increasing doses of dexamethasone given at night

Date	Condition	Cortisol (mcg/dl)	Acth (pg/ml)	Dexamethasone (ng/dl)
4/9/18	Baseline	21.4	51	–
4/10/18	After 2 mg dexamethasone	24.6	–	232
4/16/18	After 4 mg dexamethasone	14.1	–	588
4/23/18	After 8 mg dexamethasone	3.1	–	1330

To date (March 2018), she continues to have hypercortisolism without clinical stigmata of Cushing’s syndrome.

## Discussion

Our patient has had persistently elevated cortisol levels, which did not suppress with dexamethasone on multiple occasions. Over the last 10 years, she has not developed any clinical stigmata of Cushing’s syndrome. Pseudocushing’s, due to alcohol intake or depression were excluded. Although she had a history of depression, her depression during this time was well controlled. It is also unlikely that she had cyclic Cushing’s syndrome since the cortisol levels were persistently elevated over several years. We, therefore, believe that she has “glucocorticoid resistance syndrome”. However, no genetic abnormality was identified.

Primary generalized glucocorticoid resistance is a rare genetic condition characterized by generalized or partial target-tissue insensitivity to glucocorticoids. [2–6] This syndrome was first described by Vingerhoeds et al.in 1976. [7] They described a hypertensive, hypokalemic patient with high cortisol production rate with elevated plasma ACTH levels but no stigmata of Cushing’s syndrome and suggested that a possible genetic defect may be responsible for glucorticoid hyposensitivity features. Due to the decreased sensitivity of peripheral tissues to glucocorticoids, there is compensatory increase in the activity of the hypothalamic-pituitary-adrenal [HPA] axis leading to ACTH hypersecretion. [2–6] Excess ACTH secretion leads to increased secretion of cortisol and other adrenal steroids including mineralocorticoids and/or androgens. However, the clinical features of Cushing’s syndrome do not develop because of resistance to cortisol action.

The diagnostic features of cortisol resistance syndrome include elevated serum cortisol and 24-h urinary free cortisol without the features of hypercortisolism. [2–6] The circadian rhythm of ACTH and cortisol secretion and their responsiveness to stressors are preserved, though at higher concentrations, and there is resistance of the HPA axis to dexamethasone suppression. Unfortunately, in our patient a detailed evaluation for diurnal variation of cortisol levels was not done. Although the results are inconsistent there is some suggestion that her diurnal variation was maintained. In July 2012 her AM cortisol level was 26.3 mcg/dl and PM was 22.5 mcg/dl and in December 2012 the midnight and AM cortisol levels were 21.8 mcg/dl and 30.6 mcg/dl respectively. On the other hand there was no change in the cortisol levels obtained in the morning and evening in November 2012. We are unable to explain the inconsistency of these results, but believe that these results by themselves do not rule out the presence of cortisol resistance syndrome. Bone mineral density is useful in distinguishing from Cushing’s, which is preserved in patients with glucocorticoid resistance but not in Cushing’s syndrome. [8] Our patient demonstrated osteopenia, which is consistent for her age. She did not have osteoporosis, which would be expected with Cushing’s syndrome. [1] Performing 1 mg dexamethasone suppression tests in family members of the patient could also aid in the diagnosis. [8] The patient’s mother and daughters were tested, and they had normal cortisol levels.

The spectrum of clinical manifestations in patients can be quite wide. It can range from asymptomatic to mild or severe forms of hyperandrogenism (hirsutism, acne and menstrual irregularities), and /or mineralocorticoid excess (hypertension, hypokalemic alkalosis) accounting for various phenotypes. [2–6] Mild cases may present with fatigue due to inadequate effect of cortisol. This variability is explained by different degrees of glucocorticoid resistance, differences in target tissue sensitivity to adrenal steroids (glucocorticoids, mineralocorticoids and androgens) among patients and possibly different biochemical defects of glucocorticoid receptor mutations.

When initially seen in 2008, at age 41, her plasma testosterone levels were elevated and thought to be consistent with the “cortisol resistance syndrome”. However, by the year 2012 at approximately age 45, the plasma testosterone levels had significantly decreased. Since her menstrual periods were irregular, it is difficult to pinpoint the exact time of the onset of menopause. Never the less, the significant decrease of testosterone levels at age 45 suggests that the source of increased androgens may have been the ovaries rather than adrenal glands.

The molecular basis of the cortisol resistance syndrome in most cases is due to inactivating point mutations, insertions or deletions in the *NR3C1* gene; which encodes the human glucocorticoid receptor (hGR).[(2–6] This leads to a defective glucocorticoid receptor and impaired glucocorticoid signaling, thereby modifying tissue sensitivity to glucocorticoids. Both heterozygous and homozygous mutations have been described. [9]

Based on the fact that most of the mutations are heterozygous it has been suggested that that complete loss-of-function of the receptor is incompatible with life. [2] However, an Australian boy was born with a homozygous 2 base pair deletion in the ligand binding domain of the glucocorticoid receptor. He survived at least till the age of 5 months. [10] A number of patients with this disorder have been described where sequencing of the hGR gene did not reveal mutations. [11] A defect anywhere in the pathway of action of glucocortoids including, reduced number of receptors, a decrease in the ligand affinity, or a post receptor defect could explain the pathogenesis in these patients including the one described here.

In addition to sequencing the coding region of the *NR3C1* gene, other molecular biology methods like dexamethasone–binding assays and thymidine incorporation assays can be used to confirm the diagnosis of glucocorticoid resistance, but were not available to us. [5]

The goal of treatment is to suppress the excess secretion of ACTH, thereby decreasing the increased production of mineralocorticoids and androgens from the adrenal gland. [2–6] High doses of drugs like dexamethasone are effective in suppressing ACTH secretions and can be titrated based on clinical manifestations. The treatment should be individualized. Mild asymptomatic patients, such as ours may not require suppression by dexamethasone.

## Conclusion

Generalized glucocorticoid resistance is a rare disease characterized by high cortisol levels but no stigmata of Cushing’s syndrome. Only a few cases have been described so far. However, its incidence might be underestimated due to non-specific signs and symptoms. Due to inadequate negative feedback, ACTH levels rise leading to stimulation of the adrenal glands, producing supraphysiological levels of cortisol, mineralocorticoids and androgens but clinical manifestations of Cushing’s syndrome do not develop because of resistance to cortisol action. Majority of patients, but not all, have a genetic mutation in the hGR *NR3C1* gene.

Our patient had characteristic features of asymptomatic glucocorticoid resistance without an identifiable hGR mutation suggesting a variant form of this disorder.

## Abbreviations

ACTH: adrenocorticotropic hormone
BMD: bone mineral density
BMI: body mass index
DHEA-S: dehydroepiandrosterone sulfate
DST: dexamethasone suppression tests
DXA: dual-energy x-ray absorptiometry
hGR: human glucocorticoid receptor
HPA: hypothalamic-pituitary-adrenal
MRI: magnetic resonance imaging
MSU: Michigan State University
